# Supplementary material for: Underrepresentation of women in the senior levels of Brazilian science
Source: PeerJ. 2017 Dec 19;5:e4000. doi: 10.7717/peerj.4000 (PMC5741063; doi:10.7717/peerj.4000)
Supplement: Supplemental Information 7 — Analyses. [file peerj-05-4000-s007.docx]

**Gender distribution of productivity scholarship holders in sub-areas of knowledge**

**Association between gender and the sub-areas of Engineering, and Exact and Earth Sciences**

| **Subareas * Gender Tabulação cruzada** | | | | |
| --- | --- | --- | --- | --- |
| Contagem | | | | |
| Subareas | | Gender | | Total |
|  |  | Female | Male |  |
|  | Astronomy | 20 | 79 | 99 |
|  | Computer Science | 88 | 287 | 375 |
|  | Industrial Design | 14 | 16 | 30 |
|  | Aerospace Engineering | 10 | 44 | 54 |
|  | Sanitary Engineering | 36 | 88 | 124 |
|  | Biomedical Engineering | 4 | 60 | 64 |
|  | Civil Engineering | 56 | 210 | 266 |
|  | Electrical Engineering | 13 | 269 | 282 |
|  | Materials Engineering | 87 | 247 | 334 |
|  | Mechanical Engineering | 24 | 252 | 276 |
|  | Mining Engineering | 4 | 21 | 25 |
|  | Marine Engineering | 1 | 10 | 11 |
|  | Nuclear Engineering | 25 | 48 | 73 |
|  | Production Engineering | 26 | 62 | 88 |
|  | Chemical Engineering | 59 | 95 | 154 |
|  | Transportation Engineering | 13 | 39 | 52 |
|  | Physics | 101 | 806 | 907 |
|  | Mathematics | 29 | 271 | 300 |
|  | Oceanography | 33 | 85 | 118 |
|  | Probability and Statistics | 19 | 51 | 70 |
|  | Chemistry | 207 | 480 | 687 |
|  | Geosciences | 107 | 363 | 470 |
| Total | | 976 | 3883 | 4859 |

| **Subareas * Gender Tabulação cruzada** | | | | |
| --- | --- | --- | --- | --- |
| Contagem | | | | |
| Subareas | | Gender | | Total |
|  |  | Female | Male |  |
|  | Astronomy | 20 | 79 | 99 |
|  | Computer Science | 88 | 287 | 375 |
|  | Industrial Design | 14 | 16 | 30 |
|  | Aerospace Engineering | 10 | 44 | 54 |
|  | Sanitary Engineering | 36 | 88 | 124 |
|  | Biomedical Engineering | 4 | 60 | 64 |
|  | Civil Engineering | 56 | 210 | 266 |
|  | Electrical Engineering | 13 | 269 | 282 |
|  | Materials Engineering | 87 | 247 | 334 |
|  | Mechanical Engineering | 24 | 252 | 276 |
|  | Mining Engineering | 4 | 21 | 25 |
|  | Marine Engineering | 1 | 10 | 11 |
|  | Nuclear Engineering | 25 | 48 | 73 |
|  | Production Engineering | 26 | 62 | 88 |
|  | Chemical Engineering | 59 | 95 | 154 |
|  | Transportation Engineering | 13 | 39 | 52 |
|  | Physics | 101 | 806 | 907 |
|  | Mathematics | 29 | 271 | 300 |
|  | Oceanography | 33 | 85 | 118 |
|  | Probability and Statistics | 19 | 51 | 70 |
|  | Chemistry | 207 | 480 | 687 |
|  | Geosciences | 107 | 363 | 470 |
| Total | | 976 | 3883 | 4859 |

| **Testes de qui-quadrado** | | | |
| --- | --- | --- | --- |
|  | Valor | df | Sig. Assint. (2 lados) |
| Qui-quadrado de Pearson | 267,054^a^ | 21 | ,000 |
| Razão de verossimilhança | 284,369 | 21 | ,000 |
| Associação Linear por Linear | 3,151 | 1 | ,076 |
| N de Casos Válidos | 4859 |  |  |
| a. 1 células (2,3%) esperam contagem menor do que 5. A contagem mínima esperada é 2,21. | | | |

**Sub-areas of Life Sciences**

| **Subarea * Gender Tabulação cruzada** | | | | |
| --- | --- | --- | --- | --- |
| Contagem | | | | |
| Subarea | | Gender | | Total |
|  |  | Female | Male |  |
|  | Agronomy | 157 | 604 | 761 |
|  | Aquaculture | 22 | 47 | 69 |
|  | Biophysics | 24 | 59 | 83 |
|  | General biology | 4 | 1 | 5 |
|  | Biochemistry | 113 | 119 | 232 |
|  | Botanics | 115 | 95 | 210 |
|  | Food Science and Technology | 99 | 82 | 181 |
|  | Ecology | 68 | 126 | 194 |
|  | Physical education | 14 | 70 | 84 |
|  | Nursing | 165 | 8 | 173 |
|  | Agricultural Engineering | 17 | 127 | 144 |
|  | Pharmacy | 88 | 68 | 156 |
|  | Pharmacology | 102 | 87 | 189 |
|  | Physiology | 86 | 92 | 178 |
|  | Physiotherapy | 43 | 23 | 66 |
|  | Phonoaudiology | 50 | 1 | 51 |
|  | Genetics | 134 | 115 | 249 |
|  | Immunology | 90 | 69 | 159 |
|  | Medicine | 205 | 333 | 538 |
|  | Veterinary Medicine | 91 | 208 | 299 |
|  | Microbiology | 105 | 82 | 187 |
|  | Morphology | 64 | 52 | 116 |
|  | Nutrition | 54 | 27 | 81 |
|  | Odontology | 82 | 129 | 211 |
|  | Parasitology | 66 | 77 | 143 |
|  | Forest Engineering | 26 | 121 | 147 |
|  | Fisheries Engineering | 28 | 79 | 107 |
|  | Public Health | 114 | 85 | 199 |
|  | Zoology | 64 | 157 | 221 |
|  | Zootechnics | 59 | 195 | 254 |
| Total | | 2349 | 3338 | 5687 |

| **Subarea * Gender Tabulação cruzada** | | | |
| --- | --- | --- | --- |
| Residual ajustado | | | |
| Subarea | | Gender | |
|  |  | Female | Male |
|  | Agronomy | -12,4 | 12,4 |
|  | Aquaculture | -1,6 | 1,6 |
|  | Biophysics | -2,3 | 2,3 |
|  | General biology | 1,8 | -1,8 |
|  | Biochemistry | 2,3 | -2,3 |
|  | Botanics | 4,0 | -4,0 |
|  | Food Science and Technology | 3,7 | -3,7 |
|  | Ecology | -1,8 | 1,8 |
|  | Physical education | -4,6 | 4,6 |
|  | Nursing | 14,7 | -14,7 |
|  | Agricultural Engineering | -7,3 | 7,3 |
|  | Pharmacy | 3,9 | -3,9 |
|  | Pharmacology | 3,6 | -3,6 |
|  | Physiology | 1,9 | -1,9 |
|  | Physiotherapy | 4,0 | -4,0 |
|  | Phonoaudiology | 8,3 | -8,3 |
|  | Genetics | 4,1 | -4,1 |
|  | Immunology | 4,0 | -4,0 |
|  | Medicine | -1,6 | 1,6 |
|  | Veterinary Medicine | -3,9 | 3,9 |
|  | Microbiology | 4,2 | -4,2 |
|  | Morphology | 3,1 | -3,1 |
|  | Nutrition | 4,7 | -4,7 |
|  | Odontology | -,7 | ,7 |
|  | Parasitology | 1,2 | -1,2 |
|  | Forest Engineering | -5,9 | 5,9 |
|  | Fisheries Engineering | -3,2 | 3,2 |
|  | Public Health | 4,7 | -4,7 |
|  | Zoology | -3,8 | 3,8 |
|  | Zootechnics | -6,0 | 6,0 |

| **Testes de qui-quadrado** | | | |
| --- | --- | --- | --- |
|  | Valor | df | Sig. Assint. (2 lados) |
| Qui-quadrado de Pearson | 788,701^a^ | 29 | ,000 |
| Razão de verossimilhança | 856,880 | 29 | ,000 |
| Associação Linear por Linear | 4,383 | 1 | ,036 |
| N de Casos Válidos | 5687 |  |  |
| a. 2 células (3,3%) esperam contagem menor do que 5. A contagem mínima esperada é 2,07. | | | |

**Association between gender and the sub-areas of Humanities**

| **Subarea * Sexo Tabulação cruzada** | | | | |
| --- | --- | --- | --- | --- |
| Contagem | | | | |
| Subarea | | Sexo | | Total |
|  |  | Female | Male |  |
|  | Management | 50 | 126 | 176 |
|  | Anthropology | 74 | 66 | 140 |
|  | Archaeology | 23 | 19 | 42 |
|  | Architecture and Urbanism | 54 | 42 | 96 |
|  | Art | 61 | 42 | 103 |
|  | Information Science | 35 | 10 | 45 |
|  | Political Science | 42 | 77 | 119 |
|  | Communication | 61 | 61 | 122 |
|  | Law | 26 | 42 | 68 |
|  | Economics | 29 | 178 | 207 |
|  | Education | 242 | 136 | 378 |
|  | Philosophy | 22 | 111 | 133 |
|  | Geography | 40 | 51 | 91 |
|  | History | 113 | 125 | 238 |
|  | Letters | 126 | 102 | 228 |
|  | Linguistics | 152 | 59 | 211 |
|  | Museology | 1 | 4 | 5 |
|  | Urban Planning | 44 | 33 | 77 |
|  | Psychology | 175 | 138 | 313 |
|  | Social Service | 62 | 9 | 71 |
|  | Sociology | 88 | 106 | 194 |
|  | Theology | 2 | 5 | 7 |
|  | Tourism | 8 | 6 | 14 |
|  | Domestic Economy | 1 | 0 | 1 |
| Total | | 1531 | 1548 | 3079 |

| **Subarea * Sexo Tabulação cruzada** | | | |
| --- | --- | --- | --- |
| Residual ajustado | | | |
| Subarea | | Sexo | |
|  |  | Female | Male |
|  | Management | -5,8 | 5,8 |
|  | Anthropology | ,8 | -,8 |
|  | Archaeology | ,7 | -,7 |
|  | Architecture and Urbanism | 1,3 | -1,3 |
|  | Art | 2,0 | -2,0 |
|  | Information Science | 3,8 | -3,8 |
|  | Political Science | -3,2 | 3,2 |
|  | Communication | ,1 | -,1 |
|  | Law | -1,9 | 1,9 |
|  | Economics | -10,6 | 10,6 |
|  | Education | 5,9 | -5,9 |
|  | Philosophy | -7,8 | 7,8 |
|  | Geography | -1,1 | 1,1 |
|  | History | -,7 | ,7 |
|  | Letters | 1,7 | -1,7 |
|  | Linguistics | 6,7 | -6,7 |
|  | Museology | -1,3 | 1,3 |
|  | Urban Planning | 1,3 | -1,3 |
|  | Psychology | 2,3 | -2,3 |
|  | Social Service | 6,4 | -6,4 |
|  | Sociology | -1,3 | 1,3 |
|  | Theology | -1,1 | 1,1 |
|  | Tourism | ,6 | -,6 |
|  | Domestic Economy | 1,0 | -1,0 |

| **Testes de qui-quadrado** | | | |
| --- | --- | --- | --- |
|  | Valor | df | Sig. Assint. (2 lados) |
| Qui-quadrado de Pearson | 360,060^a^ | 23 | ,000 |
| Razão de verossimilhança | 386,989 | 23 | ,000 |
| Associação Linear por Linear | 6,837 | 1 | ,009 |
| N de Casos Válidos | 3079 |  |  |
| a. 6 células (12,5%) esperam contagem menor do que 5. A contagem mínima esperada é ,50. | | | |

**Distribution of productivity scholarship holders by gender and scholarship level**

**Association between gender and scholarship level in Exact Sciences**

| **Level * Gender Tabulação cruzada** | | | | |
| --- | --- | --- | --- | --- |
| Contagem | | | | |
|  | | Gender | | Total |
|  |  | Female | Male |  |
| Level | PQ-1A | 41 | 378 | 419 |
|  | PQ-1B | 75 | 391 | 466 |
|  | PQ-1C | 78 | 380 | 458 |
|  | PQ-1D | 161 | 595 | 756 |
|  | PQ-2 | 621 | 2139 | 2760 |
| Total | | 976 | 3883 | 4859 |

| **Level * Gender Tabulação cruzada** | | | |
| --- | --- | --- | --- |
| Residual ajustado | | | |
|  | | Gender | |
|  |  | Female | Male |
| Level | PQ-1A | -5,5 | 5,5 |
|  | PQ-1B | -2,3 | 2,3 |
|  | PQ-1C | -1,7 | 1,7 |
|  | PQ-1D | ,9 | -,9 |
|  | PQ-2 | 4,8 | -4,8 |

| **Testes de qui-quadrado** | | | |
| --- | --- | --- | --- |
|  | Valor | df | Sig. Assint. (2 lados) |
| Qui-quadrado de Pearson | 45,696^a^ | 4 | ,000 |
| Razão de verossimilhança | 50,763 | 4 | ,000 |
| N de Casos Válidos | 4859 |  |  |
| a. 0 células (0,0%) esperam contagem menor do que 5. A contagem mínima esperada é 84,16. | | | |

**Association between gender and scholarship level in Life Sciences**

| **Level * Gender Tabulação cruzada** | | | | |
| --- | --- | --- | --- | --- |
| Contagem | | | | |
| Level | | Gender | | Total |
|  |  | Female | Male |  |
|  | PQ-1A | 132 | 393 | 525 |
|  | PQ-1B | 172 | 343 | 515 |
|  | PQ-1C | 248 | 351 | 599 |
|  | PQ-1D | 418 | 580 | 998 |
|  | PQ-2 | 1379 | 1671 | 3050 |
| Total | | 2349 | 3338 | 5687 |

| **Level * Gender Tabulação cruzada** | | | |
| --- | --- | --- | --- |
| Residual ajustado | | | |
| Level | | Gender | |
|  |  | Female | Male |
|  | PQ-1A | -7,9 | 7,9 |
|  | PQ-1B | -3,8 | 3,8 |
|  | PQ-1C | ,1 | -,1 |
|  | PQ-1D | ,4 | -,4 |
|  | PQ-2 | 6,4 | -6,4 |

| **Testes de qui-quadrado** | | | |
| --- | --- | --- | --- |
|  | Valor | df | Sig. Assint. (2 lados) |
| Qui-quadrado de Pearson | 89,201^a^ | 4 | ,000 |
| Razão de verossimilhança | 92,922 | 4 | ,000 |
| N de Casos Válidos | 5687 |  |  |
| a. 0 células (0,0%) esperam contagem menor do que 5. A contagem mínima esperada é 212,72. | | | |

**Association between gender and scholarship level in Humanities**

| **Level * Gender Tabulação cruzada** | | | | |
| --- | --- | --- | --- | --- |
| Contagem | | | | |
| Level | | Gender | | Total |
|  |  | Female | Male |  |
|  | PQ-1A | 126 | 143 | 269 |
|  | PQ-1B | 158 | 132 | 290 |
|  | PQ-1C | 154 | 113 | 267 |
|  | PQ-1D | 219 | 262 | 481 |
|  | PQ-2 | 874 | 898 | 1772 |
| Total | | 1531 | 1548 | 3079 |

| **Level * Gender Tabulação cruzada** | | | |
| --- | --- | --- | --- |
| Residual ajustado | | | |
| Level | | Gender | |
|  |  | Female | Male |
|  | PQ-1A | -1,0 | 1,0 |
|  | PQ-1B | 1,7 | -1,7 |
|  | PQ-1C | 2,7 | -2,7 |
|  | PQ-1D | -2,0 | 2,0 |
|  | PQ-2 | -,5 | ,5 |

| **Testes de qui-quadrado** | | | |
| --- | --- | --- | --- |
|  | Valor | df | Sig. Assint. (2 lados) |
| Qui-quadrado de Pearson | 13,777^a^ | 4 | ,008 |
| Razão de verossimilhança | 13,811 | 4 | ,008 |
| N de Casos Válidos | 3079 |  |  |
| a. 0 células (0,0%) esperam contagem menor do que 5. A contagem mínima esperada é 132,76. | | | |

**Brazilian Academy of Science**

| **Gender** | | | | | |
| --- | --- | --- | --- | --- | --- |
|  | | Frequência | Porcentual | Porcentagem válida | Porcentagem acumulativa |
|  | Female | 126 | 14,0 | 14,0 | 14,0 |
|  | Male | 773 | 86,0 | 86,0 | 100,0 |
|  | Total | 899 | 100,0 | 100,0 |  |

| **AreaNUM * Gender Tabulação cruzada** | | | | |
| --- | --- | --- | --- | --- |
| % dentro de AreaNUM | | | | |
|  | | Gender | | Total |
|  |  | Female | Male |  |
| AreaNUM | Exact Sciences | 8,9% | 91,1% | 100,0% |
|  | Life Sciences | 20,4% | 79,6% | 100,0% |
|  | Humanities | 18,2% | 81,8% | 100,0% |
| Total | | 14,0% | 86,0% | 100,0% |

| **Testes de qui-quadrado** | | | |
| --- | --- | --- | --- |
|  | Valor | df | Sig. Assint. (2 lados) |
| Qui-quadrado de Pearson | 23,875^a^ | 2 | ,000 |
| Razão de verossimilhança | 23,873 | 2 | ,000 |
| Associação Linear por Linear | 20,199 | 1 | ,000 |
| N de Casos Válidos | 899 |  |  |
| a. 1 células (16,7%) esperam contagem menor do que 5. A contagem mínima esperada é 4,63. | | | |

**Amount of funding awarded by gender**

Amount of funding awarded by gender in the UNIVERSAL CNPQ CALL

| **RangeFunding * Gender Tabulação cruzada** | | | | |
| --- | --- | --- | --- | --- |
| Contagem | | | | |
| RangeFunding | | Gender | | Total |
|  |  | Male | Female |  |
|  | 60-120 thousand Brazilian reais | 407 | 175 | 582 |
|  | 30-60 thousand Brazilian reais | 671 | 398 | 1069 |
|  | < 30 thousand Brazilian reais | 1288 | 897 | 2185 |
| Total | | 2366 | 1470 | 3836 |

| **RangeFunding * Gender Tabulação cruzada** | | | |
| --- | --- | --- | --- |
| Residual ajustado | | | |
| RangeFunding | | Gender | |
|  |  | Male | Female |
|  | 60-120 thousand Brazilian reais | 4,4 | -4,4 |
|  | 30-60 thousand Brazilian reais | ,9 | -,9 |
|  | < 30 thousand Brazilian reais | -4,0 | 4,0 |

| **Testes de qui-quadrado** | | | |
| --- | --- | --- | --- |
|  | Valor | df | Sig. Assint. (2 lados) |
| Qui-quadrado de Pearson | 24,204^a^ | 2 | ,000 |
| Razão de verossimilhança | 24,714 | 2 | ,000 |
| Associação Linear por Linear | 23,400 | 1 | ,000 |
| N de Casos Válidos | 3836 |  |  |
| a. 0 células (0,0%) esperam contagem menor do que 5. A contagem mínima esperada é 223,03. | | | |
